# RE: Combined serological tests for pulmonary aspergillosis

**DOI:** 10.4103/0256-4947.72273

**Published:** 2010

**Authors:** Viroj Wiwanitkit

**Affiliations:** Wiwanitkit House, Bangkhae, Bangkok 10160, Thailand wviroj@yahoo.com

**To the Editor**: I read the recent publication by Gao et al on combined serological tests for pulmonary aspergillosis with a great interest.^1^ Gao et al concluded that “Combining two serological tests increased the specificity of diagnosis, but further trials are needed to prove the value of this approach.”^1^ First, clarification of the quality control process for the serological test in this study is necessary since this can affect interpretation of the results. In addition, I would like to add some ideas on this report. In addition to a trial on the diagnostic properties in other settings, a study on the cost effectiveness of this new approach should also be conducted. Also, there should be a diagnostic protocol for management of cases with controversial and discordant results from the combined serological tests. Indeed, the problem of cross reaction to other nonpathological *Aspergillus* spp should also be kept in mind when one uses the serological test for diagnosis.^2^ However, Lau et al suggested that “Notwithstanding the advantages of molecular tests, serological assays remain clinically useful for patient management.”^3^
